# Ultra-strong stability of double-sided fluorinated monolayer graphene and its electrical property characterization

**DOI:** 10.1038/s41598-020-74618-4

**Published:** 2020-10-16

**Authors:** Haidong Wang, Masahiro Narasaki, Zhongwei Zhang, Koji Takahashi, Jie Chen, Xing Zhang

**Affiliations:** 1 Department of Engineering Mechanics, Tsinghua University, Beijing, 100084 People’s Republic of China; 2 Department of Aeronautics and Astronautics, Kyushu University, 744 Motooka, Fukuoka, 819-0395 Japan; 3 International Institute for Carbon-Neutral Energy Research (WPI-I2CNER), Kyushu University, 744 Motooka, Fukuoka, 819-0395 Japan; 4 Center for Phononics and Thermal Energy Science, China–EU Joint Lab for Nanophononics, School of Physics Science and Engineering, Tongji University, Shanghai, 200092 People’s Republic of China

**Keywords:** Graphene, Nanoscale devices

## Abstract

Fluorinated graphene has a tunable band gap that is useful in making flexible graphene electronics. But the carbon–fluorine (C–F) bonds in fluorinated graphene can be easily broken by increased temperature or electron beam irradiation. Here, we demonstrate that the stability of fluorinated graphene is mainly determined by its C–F configuration. The double-sided fluorinated graphene has a much stronger stability than the single-sided fluorinated graphene under the same irradiation dose. Density functional theory calculations show that the configuration of double-sided fluorinated graphene has a negative and low formation energy, indicating to be an energetically stable structure. On the contrary, the formation energy of single-sided fluorinated graphene is positive, leading to an unstable C–F bonding that is easily broken by the irradiation. Our findings make a new step towards a more stable and efficient design of graphene electronic devices.

## Introduction

Graphene has attracted intense attention since its discovery^1^. Utilizing the superior properties of this two-dimensional (2D) material, such as the exceptionally high charge mobility^2^, high optical transparency^3^, excellent flexibility^4^, ultrahigh mechanical strength and thermal conductivity^5–9^, pristine graphene can be used in a wide range of applications. However, the special feature of zero bandgap in graphene has greatly limited its application as a semiconductor^10–12^. Consequently, it is important to open the bandgap of pristine graphene through the bandgap engineering and explore its utilization as a novel 2D semiconductor. Fluorination has been proved to be an efficient method to modify the electronic structures of graphene. The band gap in the fully fluorinated graphene (F/C ratio is 50%) can be opened up to 3.07 eV based on the theoretical calculation^13^. The rise of fluorinated graphene makes the flexible and transparent all-graphene-electronics possible^14^. By simply changing the fluorine/carbon (F/C) ratio of this atomic layer, the electrical characteristic of fluorinated graphene can be tuned from conductive, semiconductive to insulating. The electron beam lithography (EBL) and direct electron beam irradiation have been practically used to open the nanometer-sized conductive channels in the fluorinated graphene and fabricate the all-graphene-electronic devices^14–16^.

Synthesis of fluorinated graphene and its stability are important for designing the flexible and printed electronic devices^17–20^. The conventional synthesis methods include chemical fluorination and physical exfoliation: the former includes direct gas fluorination^13,21^, plasma fluorination^22–24^, hydrothermal fluorination^25^, etc.; the latter includes sonochemical exfoliation^26,27^, modified Hummer’s exfoliation^28^, thermal exfoliation^29^, etc. The F/C ratio in the fluorinated graphene depends on the fluorine agents or exfoliation solvents, the reaction temperature and time. By using the same synthetic method, higher reaction temperature and longer reaction time will result in a higher F/C ratio.

So far, there are a lot of works focused on the synthesis of the fluorinated graphene, while less attention has been paid to the stability of fluorinated graphene. Recently, researchers found that the stability of fluorinated graphene is sensitive to both temperature^30,31^ and electron beam irradiation^13,14^. A short time of annealing at temperatures below 400 °C or low-dose electron beam irradiation will partially recover fluorinated graphene to pristine graphene. A direct proof for the recovery of fluorinated graphene is the decrease in the D-band Raman spectra and significant increase in the electrical conductivity. Prolonged annealing time at high temperature and high-dose electron beam irradiation may remove both C and F atoms in the fluorinated graphene^14,31^. On the other hand, single-sided fluorinated graphene with low F/C ratio prepared by using XeF_2_ gas will lose 50–80% initial fluorine coverage over 10 days before reaching a saturated state^32^. All these facts show that the fluorinated graphene possesses a poor stability over storage time, temperature and irradiation, which severely limits the practical utilization of fluorinated graphene as a candidate of 2D semiconductor. It is interesting to know that the stability of fluorinated graphene is related to the F/C ratio of sample. A highly fluorinated graphene on substrate remains insulating after 10 days while a mildly fluorinated sample is partially recovered to its pristine state. It reflects the fact that stability of fluorinated graphene depends on the site and structure of C–F bonds, but the physical mechanism remains unclear. Understanding the stability of C–F bonding at molecular level and its electrical property is the key for the practical design of fluorinated graphene-based 2D semiconductors.

In this work, we have synthesized both single-sided and double-sided fluorinated monolayer graphene by using XeF_2_ gas. Subsequently, the electron beam irradiation was used to selectively remove the fluorine atoms on graphene, and the resultant variation of the sample’s electrical conductivity was monitored continuously. The measurement results show that the electrical conductivity of double-sided fluorinated graphene remained unchanged after electron beam irradiation, while the electrical conductivity of single-sided sample was significantly increased due to the loss of fluorine atoms. Such comparison demonstrates that the structure of C–F bonding at both sides of graphene has much stronger stability than that at the single side. First-principle calculations further confirmed the symmetric charge distribution and stable formation energy in the double-sided fluorinated graphene, which explains the strong stability of double-sided C–F bonding at molecular level. This work highlights the importance of double-sided fluorination to achieve stable chemically functionalized graphene samples, which can be used in the practical design of all-graphene transparent and flexible electronics.

## Synthesis of fluorinated graphene

**Figure 1:**
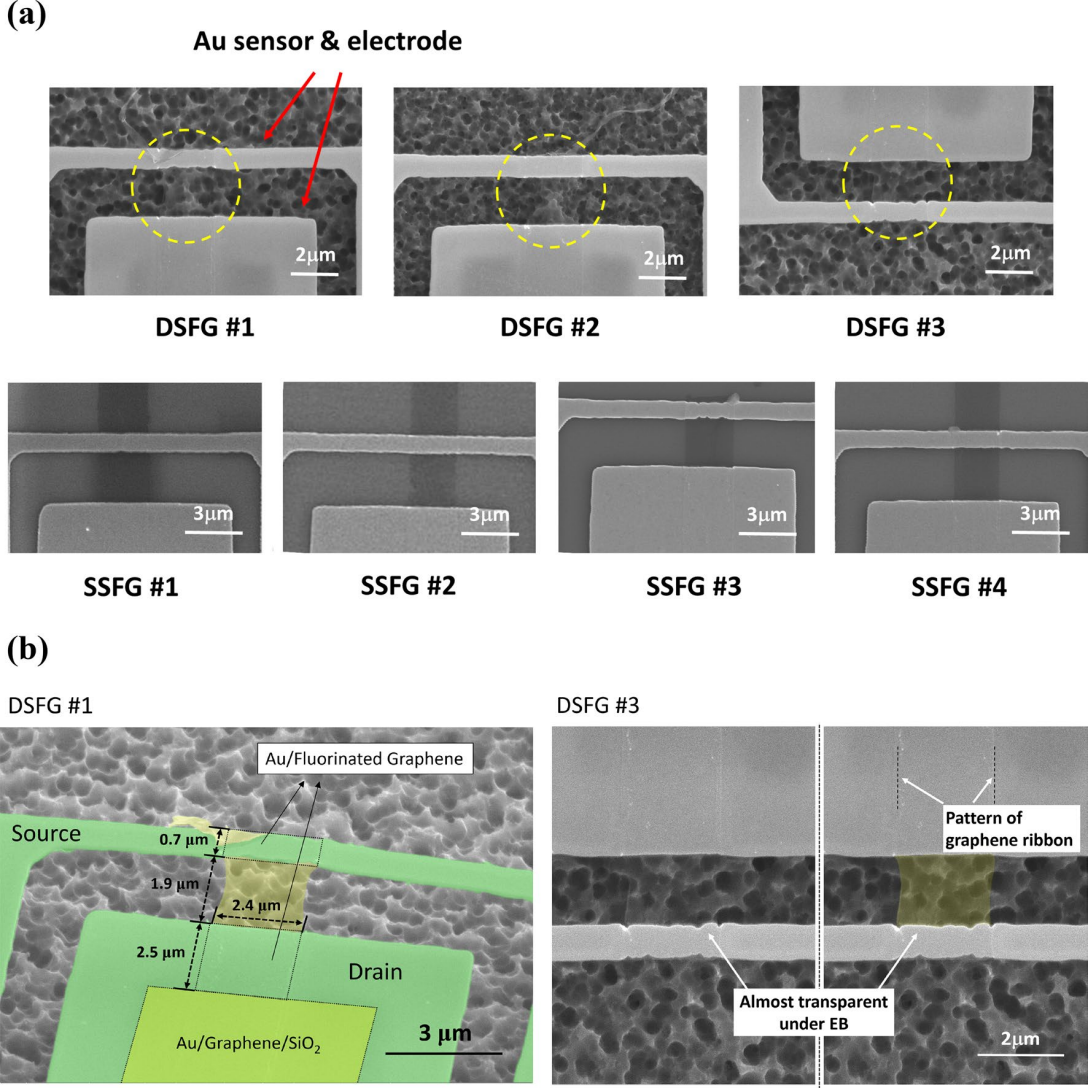
SEM images of suspended and supported fluorinated graphene samples. (**a**) SEM images of all samples, where the yellow dashed circles highlight the free-standing fluorinated graphene ribbon, which is almost transparent under the electron beam; (**b**) Zoom-in image of DSFG sample and its geometric sizes.

The fabrication process of fluorinated graphene is given as following^30,31^. At first, a monolayer graphene was grown on copper foil by using a conventional chemical vapor deposition method. Then, a thin layer of polymethyl methacrylate (PMMA) was spin-coated on the surface and used as supporting layer during the transfer process. The copper foil was etched away and the graphene/PMMA layer was transferred onto a SiO_2_/Si substrate. After that, the graphene was cut into micro-ribbons following the EBL patterning and O_2_ plasma etching. In order to measure the electrical properties of functionalized graphene sample, a thin metallic film (10 nm thick Cr and 100 nm thick Au) was deposited on the graphene ribbon by using an electron beam physical vapor deposition method. The patterned metallic electrodes were made by using EBL and a standard lift-off method. Then, the graphene was chemically modified following two schedules: (1) For the single-sided fluorinated graphene, the graphene supported on SiO_2_/Si substrate was directly put into a XeF_2_ gas etcher (BP-3F Samco Inc.). The thin SiO_2_ passivation layer blocked the contact between XeF_2_ gas and backside of graphene, thus only the top surface of graphene was fluorinated in the XeF_2_ gas. (2) For the double-sided fluorinated graphene, the thin SiO_2_ layer was removed in a buffered hydrofluoric solution. Then, the graphene ribbon partially supported on silicon was put into the XeF_2_ gas etcher and the silicon substrate was etched by a depth around 8 μm. Finally, the graphene ribbon was suspended between two metallic electrodes above the substrate. In this way, both top and bottom surfaces of graphene were fluorinated in the XeF_2_ gas. The fluorination of graphene was done at room temperature. The F/C ratio of fluorinated graphene can be tuned by controlling the exposure time in the XeF_2_ gas. Scanning electron microscope (SEM) images of tested samples can be found in Fig. 1.

Figure 1a shows 3 samples of double-sided fluorinated graphene (DSFG) and 4 samples of single-sided fluorinated graphene (SSFG). Both DSFG and SSFG samples are bridged between Au sensor and electrode (marked by the red arrows). It is worth noting that both electrical and thermal contact between graphene and the directly deposited metallic thin film are much better than those of the conventional method by transferring graphene onto the fabricated electrodes. No residual air bubbles, contaminations or wrinkles were trapped between the metal and graphene^33–37^. In the fabrication process, the SiO_2_ layer was removed by using a buffered hydrofluoric acid (BHF) through a micrometer sized window opened in the polymer resist layer. Due to the isotropic etching behavior of BHF, a part of SiO_2_ below graphene and metal was removed. The length of over-etching was measured to be 2.5 μm in the SEM image of device taken after released from the silicon substrate by using XeF_2_ etching. So it is seen in Fig. 1 that the SiO_2_ under the metallic source, SiO_2_ between source and drain, and a 2.5 μm wide SiO_2_ section under the metallic drain were removed. But SiO_2_ in the middle of the drain pad remains and forms an Au/Graphene/SiO_2_ sandwich structure, shown as the light green part in the figure. XeF_2_ gas cannot permeate the SiO_2_ layer, so the graphene between Au and SiO_2_ still remains its pristine crystalline structure. In Fig. 1b, the graphene ribbon in two square areas marked by dashed lines was fluorinated at the bottom surface, while the graphene ribbon marked by yellow color between source and drain was fluorinated at both surfaces. It was reported that the absorption spectra of graphene were drastically changed after fluorination. The fully fluorinated graphene (F/C ratio is 50%) has an almost zero absorption below 3 eV, and thus only the light with energy higher than 3.0 eV can be absorbed by the fluorinated graphene^38^. This indicates that the fluorinated graphene is almost transparent in the range of visible light with energy from ~ 1–3 eV^38^. The absorption spectrum of partially fluorinated graphene is higher than that of the fully fluorinated graphene, and slowly increases as the photon energy increases. Hence, the fluorinated graphene with low F/C ratio appears semi-transparent under the light. As seen in Fig. 1, the dependence of electron beam transparency on the F/C ratio is very similar to the behavior of light transparency. For the fully fluorinated graphene, i.e. DSFG, almost 100% electron beam can pass through the free-standing graphene layer at 10 kV accelerating voltage and 2.9 pA current, while for the partially fluorinated graphene, i.e. SSFG, the graphene layer is semi-transparent under the electron beam.

## Raman spectra, XPS and XRD results

Raman spectra, X-ray photoelectron spectroscopy (XPS) and X-ray diffraction (XRD) data were collected on the fabricated samples.

**Figure 2:**
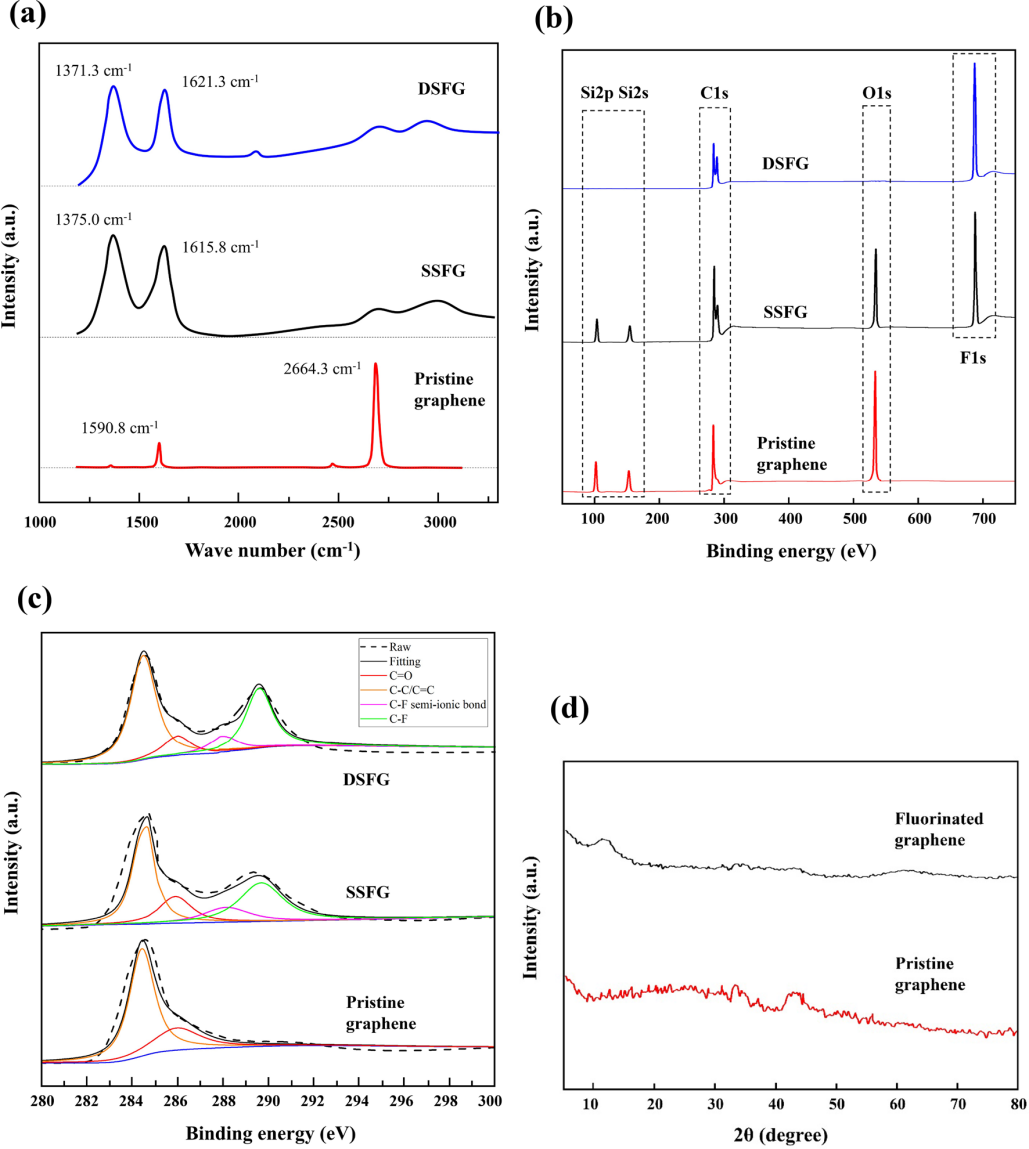
(**a**) Raman spectra, (**b**, **c**) XPS data and curve fitting, (**d**) XRD data of fluorinated and pristine graphene samples.

Figure 2a shows the Raman spectra of samples, which were taken by using T64000 system from Horiba Inc. The pristine graphene represents a typical Raman spectrum of monolayer carbon crystal, in which the intensity of 2D-band peak is much higher than that of G-band peak. For the pristine graphene, the D-band peak for defects is negligibly small. After fluorination in XeF_2_ gas on the top surface of supported graphene, the 2D-band peak is dramatically decreased, while the D-band peak increased and exceeds the G-band peak. All the Raman peaks of fluorinated graphene are widened and the baseline has been raised, comparing with the Raman spectrum of pristine graphene. These features indicate that the fluorination will break the symmetrical two-dimensional lattice structure of graphene and increase its defect level. In the case of DSFG, the baseline of Raman spectrum keeps increasing and the ratio between 2D-band and G-band peaks is further decreased. This indicates a higher level of fluorination to the graphene lattice.

Figures 2b and 2c show the XPS signals of samples which were taken by using PHI Quantera II system from Ulvac-Phi Inc. The individual spectra of C1s, O1s and F1s are shown in the figure. The strong F1s peaks in DSFG and SSFG samples indicate a high level of fluorination in graphene, while the F1s peak is missing for pristine graphene. The higher F1s peak and C-F peak in DSFG indicate a higher fluorine coverage. Based on the spectra of C1s and F1s, the atomic ratio between F and C was estimated to be around 20% and 50% for SSFG and DSFG, respectively. The atomic ratio between Si and O was calculated to be 1:2, indicating that the O and Si signals are origin from the SiO_2_ layer under graphene. The Si and O signals were missing for the suspended DSFG sample.

Figure 2d shows the XRD signals of samples which were taken by using D/Max 2500v/pc system from Japan Rigaku Inc. It is seen that the (002) peak around 25° is missing for the pristine graphene, while this peak is usually sharp and strong for the stacked graphite samples. It confirms the monolayer structure of the graphene sample. For the fluorinated graphene, a small and broad peak was noticed around 12°.

## Electrical property of fluorinated graphene

It has been reported that the electron beam irradiation can be used to remove the fluorine atoms on graphene and regain its electrical conductivity^13,14^. It is an effective and precise tool to investigate the stability of C–F bonding. In order to conduct in-situ electrical characterization of fluorinated graphene in the SEM chamber, four-lead wires were fed through to connect the graphene sample and two high-precision Keithley digital multimeters (2002 series). The *I-V* curve of fluorinated graphene was measured by using a four-probe method. Electrical measurement inside the SEM chamber (< 10^–4^ Pa) prevents contamination or dopant in the atmospheric environment, which may further change the resistance of graphene sample. A low energy electron beam (10 kV, 2.9 pA) was used for observation in the experiment, which does not significantly affect the electrical property of graphene^14^.

**Figure 3:**
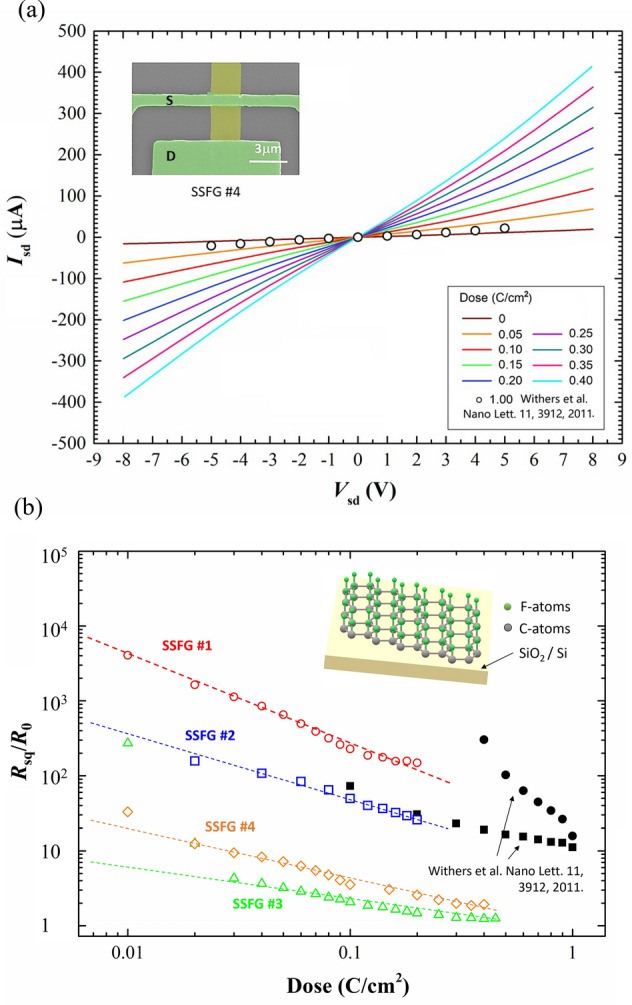
(**a**) I-V characteristics of SSFG sample #4. The electron beam irradiation dose was increased from 0 to 0.40 C/cm2. The I-V curve of fluorinated graphene reported in Ref.^14^ is shown as open circles. Inset: the SEM image of device, where the green parts are the Au film electrodes used as source and drain, respectively, the yellow part between source and drain is the fluorinated graphene ribbon. (**b**) The resistance per square of SSFG sample normalized by the reference resistance of pristine graphene. The dashed line is the guideline for eyes. The resistance of fluorinated graphene reported in Ref.^14^ is shown as black solid symbols. Inset: the illustration of single-sided fluorinated graphene supported on substrate.

Figure 3a shows the source-drain *I-V* curve of SSFG sample #4. The whole SSFG ribbon between source and drain was uniformly irradiated by an electron beam, with a dose gradually increased from 0 to 0.40 C/cm^2^. A similar experimental result^14^ of single-sided fluorinated graphene with a dose up to 1.00 C/cm^2^ is also included in Fig. 3a for comparison. The *I-V* curve of SSFG sample shows a typical symmetric behavior with respect to the bias voltage ranging from − 9 V to 9 V. Higher electron beam irradiation dose removes more fluorine atoms from graphene surface, and the source-drain current *I*_sd_ increases monotonically. Hence, the resistance per square of SSFG decreases quite rapidly with the increasing irradiation dose, as shown in Fig. 3b. It is seen that our result has the same resistance-dose declining trend as the SSFG sample reported in the literature^14^. The fluorine atoms on graphene skeleton reduce the charge in the conducting orbitals and introduce more scattering centers. Thus, the resistivity of fluorinated graphene is much larger than its pristine state, and is highly sensitive to the F/C ratio. Although four SSFG samples have almost the same geometric size and crystalline quality as a pristine graphene before fluorination, the fluctuation in the XeF_2_ gas pressure and reaction time may cause significant difference in the resistances of SSFG samples. As for SSFG #3, the resistance was decreased by 200 times after electron beam irradiation and approached a saturation value of the pristine graphene. This result demonstrates that the single-sided fluorinated graphene can be easily tuned from an insulator to a conductor by simply applying electron beam irradiation. However, the electrical property of SSFG is not so stable in two aspects: (i) the resistivity of sample is highly sensitive to the synthesis process of fluorination, and (ii) the resistivity of partially fluorinated graphene may change due to various reasons, such as irradiation, temperature, dopant in the atmospheric environment or storage time^17,32^. This can be understood by the fact that even a low energy electron beam irradiation can alter the resistance of SSFG, and the other factors (temperature, dopant, etc.) at the same energy level will have the similar impact.

**Figure 4:**
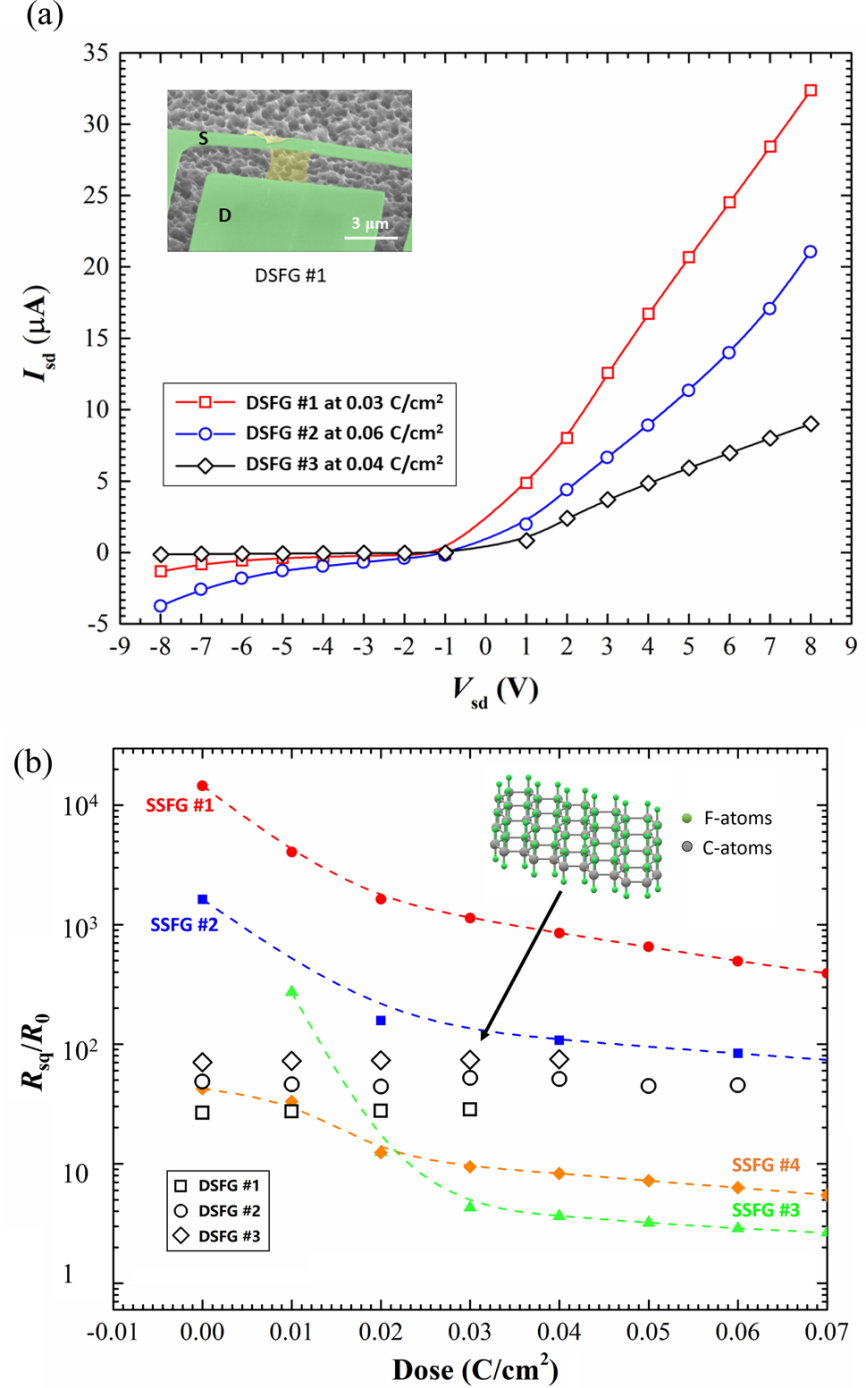
(**a**) I-V characteristics of DSFG samples. The maximum electron beam irradiation doses of three DSFG samples varies from 0.03 to 0.06 C/cm2. Inset: the SEM image of device #1, where the green parts are the Au film electrodes used as source and drain, respectively, the yellow part is the fluorinated graphene ribbon suspended between two electrodes. (**b**) The normalized resistance plotted with respect to the electron beam irradiation dose on a logarithmic scale. The black open symbols are the results of DSFG samples, while the other small solid symbols with dashed guidelines are the results of SSFG samples. Inset: the illustration of free-standing graphene with fluorine atoms bonding on two surfaces.

Figure 4 shows the source-drain *I−V* curve and resistance per square of DSFG samples. The *I−V* curve in DSFG sample represents a typical asymmetric diode characteristic, which is totally different from the metallic symmetric electrical behavior of SSFG shown in Fig. 4a. Similar to the normal graphene/doped silicon vertically stacked structure^40–43^, a charge rectification behavior is noticed in the metal/fluorinated graphene (MFG). The graphene below metallic film was fluorinated at the bottom surface with an opened band gap up to 2.8 eV^13^. The difference in the work functions between metal and fluorinated graphene causes a potential barrier at their interface, leading to the charge rectification phenomenon. Here, the thickness of MFG heterojunction is mainly decided by the metallic film, because the fluorinated graphene only has a thickness less than 1 nm. It has a potential to create a vertically stacked diode within several nanometers by reducing the thickness of metallic film. For the SSFG samples supported on SiO_2_/Si, no charge rectification behavior was shown in Fig. 3a, since the pristine graphene between metal and SiO_2_ has a zero band gap. This result proves the validity of building nanoscale diodes by using fluorinated graphene with a tunable band gap.

In addition to the charge rectification at the MFG interface, the main finding in Fig. 4b is that the resistance of DSFG is independent of the dose for electron beam irradiation, which is in sharp contrast with the exponentially decreasing resistance of SSFG with an increasing irradiation dose. This result demonstrates that the C–F bond is much stronger and more stable in DSFG than that in SSFG. The electron beam irradiation cannot dissociate the C–F bond in the free-standing F–C-F molecular structure, shown in the inset of Fig. 4b. It can be noticed that the irradiation dose for DSFG is smaller than that for SSFG. This is due to the fact that the suspended fluorinated graphene is much more vulnerable than the supported graphene when the device encounters electrical fluctuation during the measurement. Even a small voltage fluctuation could break the free-standing monolayer graphene ribbon. In the experiment, a full *I-V* curve of DSFG was measured from − 8 V to 8 V at each irradiation dose. Extreme caution should be paid to load the bias voltage on DSFG, and it took a relatively long time to obtain a single experimental data point in Fig. 4b. Unfortunately, all the three DSFG samples were broken before reaching a high irradiation dose, and all the available data are shown in Fig. 4b. However, the limited data points of DSFG do not affect the conclusion. As seen in the case of SSFG, the electron beam irradiation removes the fluorine atoms more efficiently in the beginning, and then becomes saturated as irradiation dose increases. For example, the sample SSFG #3 regained over 40% electrical conductance in the beginning of 0.06 C/cm^2^ dose irradiation. In contrast, the same electron beam irradiation didn’t cause any reduction in the resistance of DSFG, but instead slightly increased the resistance. These results all proves the fact that the C-F bonding in DSFG has a much stronger stability than that in SSFG.

## First-principle calculations

In order to understand the mechanism responsible for the ultra-strong stability observed in DSFG, first-principle calculations were carried out by using the Vienna Ab initio Simulation Package (VASP) based on density functional theory (DFT) and planewave basis^44,45^. The electron–ion interactions are described by the projector-augmented-wave (PAW) method^46^. The Perdew-Burke-Ernzerhof (PBE) functional within the generalized-gradient approximation (GGA) is used to treat the exchange–correlation interaction of electrons^47^. A kinetic energy cutoff of 400 eV for the planewave basis and a convergence criterion of 10^–7^ eV for the total energies are adopted for all ab initio calculations. The van der Waals (vdW) interaction is included according to the Tkatchenko and Scheffler method^48^.

To gain insight into the stability of fluorinated graphene, we calculated the averaged formation energy per fluorine atoms (*e*_F_) by performing ab initio calculations. Take the suspended graphene for instance, the *e*_F_ is calculated as^49^:

1 $$ e_{F} = \frac{{E_{{{\text{FG}}}} - N_{{\text{F}}} E_{{\text{F}}} - E_{{\text{G}}} }}{{N_{{\text{F}}} }} $$

where, *E*_FG_, *E*_F_ and *E*_G_ are the total energy of fluorinated graphene, one fluorine atom, and pure graphene sheet, respectively. *N*_F_ is the number of fluorine atoms in the calculation cell.

**Figure 5:**
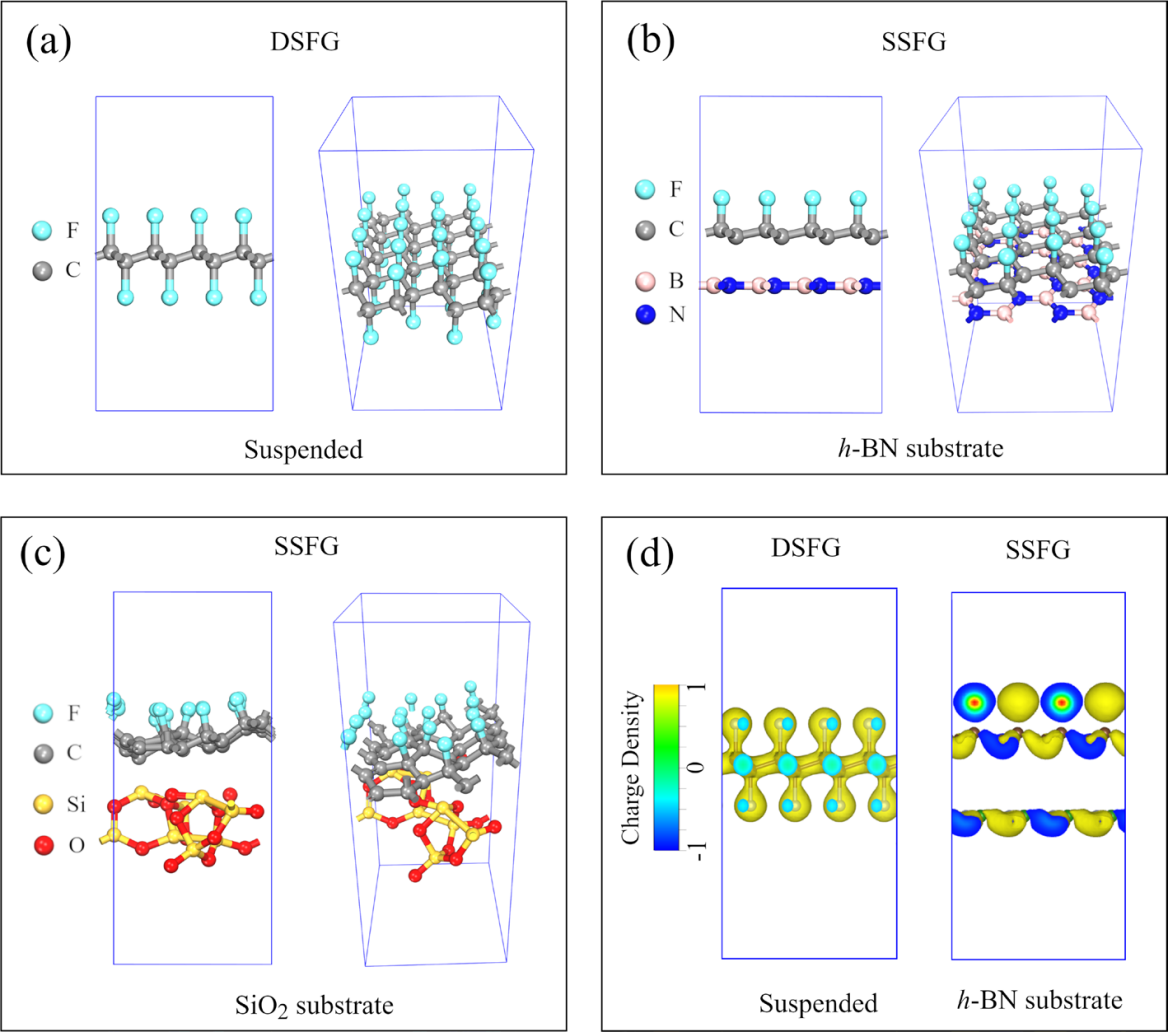
Schematic figures of fluorinated graphene in calculation. (**a**) The side view and perspective view of crystal structure of DSFG. (**b**) The side view and perspective view of crystal structure of the SSFG supported on *h*-BN substrate. (**c**) The side view and perspective view of crystal structure of the SSFG supported on amorphous SiO_2_ substrate. (**d**) Deformation charge density of the DSFG and SSFG supported on *h*-BN substrate.

For the case of DSFG, we consider the structure of the fully fluorinated graphene (F/C ratio is 100%). The optimized crystal structure is shown in Fig. 5a. Moreover, the averaged formation energy *e*_F_ is − 1.0534 eV, exhibiting a strong stability. The negative sign of *e*_F_ indicates that the adsorption of fluorine atoms on graphene would be energetically favorable. As a comparison, we further construct supported SSFG where the top surface is fluorinated while the bottom surface is supported on substrate, to mimic the experimental condition. The hexagonal boron nitride (*h*-BN) and silicon dioxide (SiO_2_) are used as the substrate in our calculations, where are two typical dielectric substrates in literatures^50,51^. The stability of SSFG supported on the substrate of *h*-BN and amorphous SiO_2_ was studied respectively. As shown in Fig. 5b and c, the surface of SSFG supported on *h*-BN substrate is relatively flat, while it is notably curved on amorphous SiO_2_ substrate. The *e*_F_ of the supported graphene was calculated similar as Eq. (3) after replacing *E*_FG_ with the total energy of fluorinated graphene with substrate, and *E*_G_ with the total energy of pure graphene with substrate. The *e*_F_ of the supported fluorinated graphene on *h*-BN and SiO_2_ substrates are 1.4854 eV and 1.5144 eV, respectively. Obviously, the positive values of *e*_F_ indicate an unstable C–F bonding. Therefore, the defluorination in the single-sided fluorinated graphene can be easily induced by the electron beam irradiation as observed in experiment. In addition, the rough surface of amorphous SiO_2_ substrate causes fluctuation in the supported SSFG structure, which makes the defluorination process even easier.

To further understand the bonding nature and stability in the fluorinated graphene, we calculated the deformation charge density (DCD)^52^. As shown in Fig. 5d, in the case of DSFG, the charges are uniformly distributed along C-F bonds, indicating a strong covalent bonding which explains the fact that no defluorination was observed in the experiment. On the contrary, the interaction between *h*-BN and graphene leads to the charges in graphene dangling on the substrate side, as shown in the DCD of Fig. 5d. Correspondingly, this charge transferring weakens the bonding strength between the carbon and fluorine atoms on the other side, and consequently leads to an unstable C-F bond formation. So the defluorination in the single-sided fluorinated graphene can be easily caused by the electron beam irradiation as observed in the experiment. Moreover, our additional first-principle calculations show that in the fluorinated graphene the C–C bonding still exhibits strong stability. For instance, the *e*_F_ between neighbor C atoms in DSFG is − 3.380 eV, which is higher than the *e*_F_ between fluorinate atoms and graphene layer (− 1.0534 eV). This calculation indicates the defluorination can be achieved without damaging the graphene layer as observed in the previous study^13^, which further makes the resistance of fluorinated graphene approaching to the value of pristine graphene during the defluorination process.

## Conclusions

Fluorination has been proved to be an efficient way to tune the band gap of graphene and control its electrical properties. Furthermore, electron beam irradiation was proved to be an efficient method in patterning the conductive channels in the fluorinated graphene, wherein the stability of fluorinated graphene is an important factor. In this report, we have fabricated electronic device with fluorinated graphene in two different configurations, double-sided fluorinated graphene and single-sided fluorinated graphene. The results show that the double-sided fluorinated graphene has an ultra-strong stability against the electron beam irradiation, while the single-sided fluorinated graphene loses the fluorine atoms easily even at a low irradiation dose. The first-principle calculations reveal that there exists a strong covalent bonding between the fluorine and carbon atoms in the double-sided fluorinated graphene, which well explains the ultra-strong stability observed in the experiment. Furthermore, a significant charge rectification behavior was observed at the interface between metal and fluorinated graphene. Our study provides important insights to the understanding of the unique chemical bonding characteristics in suspended graphene sheet, which is crucial for the fabrication of more stable and efficient graphene electronic devices.

## References

[1] Akinwande D, Huyghebaert C, Wang CH (2019). Graphene and two-dimensional materials for silicon technology. Nature.

[2] Banszerus L, Schmitz M, Engels S (2015). Ultrahigh-mobility graphene devices from chemical vapor deposition on reusable copper. Sci. Adv..

[3] Sheehy DE, Schmalian J (2009). Optical transparency of graphene as determined by the fine-structure constant. Phys. Rev. B.

[4] Akinwande D, Petrone N, Hone J (2014). Two-dimensional flexible nanoelectronics. Nat. Commun..

[5] Lee C, Wei X, Kysar JW, Hone J (2008). Measurement of the elastic properties and intrinsic strength of monolayer graphene. Science.

[6] H. I. Rasool, C. Ophus, W. S. Klug, A. Zettl, and James K. Gimzewski, Measurement of the intrinsic strength of crystalline and polycrystalline graphene, Nat. Commun. 4, 2811, 2013.

[7] Balandin AA (2011). Thermal properties of graphene and nanostructured carbon materials. Nat. Mater..

[8] Wang HD, Hu S, Takahashi K (2017). Experimental study of thermal rectification in suspended monolayer graphene. Nat. Commun..

[9] Z. Zhang, Y. Ouyang, Y. Cheng, J. Chen, N. Li, and G. Zhang, Physics reports, size-dependent phononic thermal transport in low-dimensional nanomaterials (In press) 2020.

[10] Balog R, Jørgensen B, Nilsson L (2010). Bandgap opening in graphene induced by patterned hydrogen adsorption. Nat. Mater..

[11] Dvorak M, Oswald W, Wu Z (2013). Bandgap opening by patterning graphene. Sci. Rep..

[12] Park JS, Choi HJ (2015). Band-gap opening in graphene: a reverse-engineering approach. Phys. Rev. B.

[13] Robinson JT, Burgess JS, Junkermeier CE (2010). Properties of fluorinated graphene films. Nano Lett..

[14] Withers F, Bointon TH, Dubois M (2011). Nanopatterning of fluorinated graphene by electron beam irradiation. Nano Lett..

[15] Martins SE, Withers F, Dubois M (2013). Tuning the transport gap of functionalized graphene via electron beam irradiation. New J. Phys..

[16] Li H, Daukiya L, Haldar S (2016). Site-selective local fluorination of graphene induced by focused ion beam irradiation. Sci. Rep..

[17] Feng W, Long P, Feng YY (2016). Two-dimensional fluorinated graphene: synthesis, structures, properties and applications. Adv. Sci..

[18] Ivanov AI, Nebogatikova NA, Kotin IA, Antonova IV (2017). Two-layer and composite films based on oxidized and fluorinated graphene. Phys. Chem. Chem. Phys..

[19] Antonova IV, Kurkina II, Gutakovskii AK (2019). Fluorinated graphene suspension for flexible and printed electronics: Flakes, 2D films, and heterostructures. Mat. Des..

[20] Antonova IV, Kotin IA, Kurkina II (2017). Graphene/fluorinated graphene systems for a wide spectrum of electronics application. J. Mater. Sci. Eng..

[21] Jeon K, Lee Z, Pollak E (2011). Fluorographene: a wide bandgap semiconductor with ultraviolet luminescence. ACS Nano.

[22] Baraket M, Walton SG, Lock EH (2010). The functionalization of graphene using electron-beam generated plasmas. Appl. Phys. Lett..

[23] Ho K, Liao J, Huang C (2014). One-step formation of a single atomic-layer transistor by the selective fluorination of a graphene film. Small.

[24] Sherpa SD, Levitin G, Hess DW (2012). Effect of the polarity of carbon-fluorine bonds on the work function of plasma-fluorinated epitaxial graphene. Appl. Phys. Lett..

[25] Gao X, Tang XS (2014). Effective reduction of graphene oxide thin films by a fluorinating agent: diethylaminosulfur trifluoride. Carbon.

[26] Chang H, Cheng J, Liu X (2011). Facile synthesis of wide-bandgap fluorinated graphene semiconductors. Chem. Eur. J..

[27] Gong P, Wang Z, Wang J (2012). One-pot sonochemical preparation of fluorographene and selective tuning of its fluorine coverage. J. Mater. Chem..

[28] Mathkar A, Narayanan TN, Alemany LB (2013). Synthesis of fluorinated graphene oxide and its amphiphobic properties. Part. Part. Syst. Char..

[29] Dubois M, Guérin K, Ahmad Y (2014). Thermal exfoliation of fluorinated graphite. Carbon.

[30] Ho K, Huang C, Liao J (2015). Fluorinated graphene as high performance dielectric materials and the applications for graphene nanoelectronics. Sci. Rep..

[31] Cheng LX, Jandhyala S, Mordi G (2016). Partially fluorinated graphene: structural and electrical characterization. ACS Appl. Mater. Interfaces.

[32] Stine R, Lee W, Whitener KE (2013). Chemical stability of graphene fluoride produced by exposure to XeF_2_. Nano Lett..

[33] Wang HD, Kurata K, Fukunaga T (2016). A simple method for fabricating free-standing large area fluorinated single-layer graphene with size-tunable nanopores. Carbon.

[34] Wang HD, Kurata K, Fukunaga T (2016). A general method of fabricating free-standing, monolayer graphene electronic device and its property characterization. Sens. Actuator A Phys..

[35] Diaz HC, Addou R, Batzill M (2014). Interface properties of CVD grown graphene transferred onto MoS_2_(0001). Nanoscale.

[36] Miseikis V., Xiang S. and Roddaro S. et al., Perfecting the growth and transfer of large single-crystal CVD graphene: a platform material for optoelectronic applications. In: Morandi V., Ottaviano L. (eds) *GraphITA. Carbon Nanostructures.* Springer, Cham.

[37] Sabki SN, Shamsuri SH, Fauzi SF (2017). Graphene transfer process and optimization of graphene coverage. Eur. Phys. J. Conf..

[38] Nair RR, Ren W, Jalil R (2010). Fluorographene: a two-dimensional counterpart of Teflon. Small.

[39] Wang HD, Zhang X, Takamatsu T (2016). Ultraclean suspended monolayer graphene achieved by in situ current annealing. Nanotechnology.

[40] Yang H, Heo J, Park S (2012). Graphene barristor, a triode device with a gate-controlled schottky barrier. Science.

[41] I. Jahangir, M. A. Uddin, A. K. Singh, Richardson constant and electrostatics in transfer-free CVD grown few-layer MoS_2_/graphene barristor with Schottky barrier modulation >0.6eV. Appl. Phys. Lett. 111, 142101, 2017.

[42] Bartolomeo AD (2016). Graphene Schottky diodes: an experimental review of the rectifying graphene/semiconductor heterojunction. Phys. Rep..

[43] Y. F. Liang, L. Yang, Electronic structure and optical absorption of fluorographene, in *Symposium YY - Computational Semiconductor Materials Science*, 1370, 2011.

[44] G. Kresse, J. Furthmüller, Efficient iterative schemes for ab initio total-energy calculations using a plane-wave basis set. *Phys. Rev. B Condens. Matter Mater. Phys*. **54**, 11169–11186 (1996) 10.1103/physrevb.54.11169 9984901

[45] Kresse G, Furthmüller J (1996). Efficiency of ab-initio total energy calculations for metals and semiconductors using a plane-wave basis set. Comput. Mater. Sci..

[46] Kresse G, Joubert D (1999). From ultrasoft pseudopotentials to the projector augmented-wave method. Phys. Rev. B.

[47] Perdew JP, Wang Y (1992). Accurate and simple analytic representation of the electron-gas correlation energy. Phys. Rev. B.

[48] T. Bučko, S. Lebègue, J. Hafner and J. G. Ángyán, Tkatchenko-Scheffler van der Waals correction method with and without self-consistent screening applied to solids, Phys. Rev. B - Condens. Matter Mater. Phys., **87**, 64110 (2013).

[49] Zhang H, Xie Y, Zhang Z, Zhong C, Li Y, Chen Z, Chen Y (2017). Dirac nodal lines and tilted semi-dirac cones coexisting in a striped boron sheet. J. Phys. Chem. Lett..

[50] Zhang ZW, Hu SQ, Chen J (2017). Hexagonal boron nitride: A promising substrate for graphene with high heat dissipation. Nanotechnology.

[51] Chen J, Zhang G, Li BW (2013). Substrate coupling suppresses size dependence of thermal conductivity in supported graphene. Nanoscale.

[52] Zhang Z, Xie Y, Peng Q, Chen Y (2016). Phonon transport in single-layer boron nanoribbons. Nanotechnology.

